# IL-37 and IL-38 in sepsis: immune-metabolic rheostats in a dynamic inflammatory continuum

**DOI:** 10.3389/fimmu.2026.1869119

**Published:** 2026-08-10

**Authors:** Hailong Yan, Lintong Ma, Xiujuan Huang, Xingbo Dang, Longyang Ma, Wei Dai, Wensheng Gao, Weizhou He, Shisan Bao, Gongliang Du

**Affiliations:** 1 Department of Emergency Surgery, Shaanxi Provincial People’s Hospital, Xi’an, Shaanxi, China; 2 Department of Haematology, 3201 Hospital, Hanzhong, Shaanxi, China; 3 Department of Haematology, Shaanxi Provincial People’s Hospital, Xi’an, Shaanxi, China; 4 Centre for Evidence-based Medicine, Gansu University of Chinese Medicine, Lanzhou, Gansu, China

**Keywords:** cytokine storm, IL-37, IL-38, intergrade, sepsis

## Abstract

Sepsis is a life-threatening syndrome driven by a dysregulated host response to infection, characterised by dynamic and overlapping phases of hyperinflammation and immune suppression. Despite advances in critical care, effective immunomodulatory therapies remain lacking, reflecting an incomplete understanding of its complex immunopathology. Central to sepsis progression is the cytokine storm, a transient but dominant inflammatory surge that contributes to endothelial injury, microvascular dysfunction, and multi-organ failure, followed in some patients by compensatory immunosuppression. Within this evolving immunological landscape, IL-37 and IL-38 have emerged as key members of the IL-1 cytokine family with context-dependent immunoregulatory functions. IL-37 broadly suppresses NF-κB- and MAPK-mediated inflammatory signalling and reprogrammes immunometabolism *via* mTOR inhibition, thereby attenuating pro-inflammatory cytokine production and modulating innate immune cell activity. IL-38, in contrast, exerts protective effects through regulation of inflammasome activation, promotion of regulatory T (Treg) cell responses, and suppression of effector T cell differentiation, collectively contributing to improved bacterial clearance and reduced systemic inflammation. Although both cytokines are elevated in sepsis and generally associated with disease severity, experimental evidence suggests that they function as endogenous brakes that limit excessive immune activation. However, clinical data remain heterogeneous, particularly regarding IL-10 regulation and stage-specific effects, highlighting context-dependent and compartment-specific actions. This mini-review synthesises current evidence on IL-37 and IL-38 in sepsis, emphasising their roles as integrated immunometabolic regulators rather than phase-specific mediators. We further discuss translational limitations and propose future directions centred on longitudinal immune profiling and precision immunomodulation.

## Introduction

Sepsis remains a leading cause of mortality among critically ill patients worldwide, driven by a dysregulated host response to infection that culminates in life-threatening organ dysfunction (1, 2). It arises when invading pathogens, often characterised by high virulence and/or a substantial microbial burden, overcome host defence mechanisms, particularly in the context of impaired barrier integrity or pre-existing host vulnerability (3, 4).

Although advances in critical care have improved short-term survival, targeted immunotherapies have largely failed to translate into clinical benefit. This likely reflects an incomplete understanding of sepsis immunopathology and its marked temporal and inter-individual heterogeneity (5, 6). Early models framed sepsis predominantly as a state of uncontrolled hyperinflammation. It is now clear, however, that pro-inflammatory and anti-inflammatory pathways are activated in parallel from the onset of infection and evolve dynamically over time, shaping both tissue injury and host defence capacity (4, 7).

In the early phase, this imbalance manifests as a cytokine storm, characterised by excessive and self-amplifying production of inflammatory mediators. This hypercytokinaemia contributes to endothelial dysfunction, microvascular leakage, and progressive organ injury (8, 9). Rather than representing a uniform or isolated stage, the cytokine storm is increasingly understood as a transient but functionally dominant inflammatory surge embedded within a broader and continuously shifting immune trajectory (10, 11).

As disease progresses, compensatory immunoregulatory mechanisms become increasingly prominent, giving rise to a state of immune suppression in a subset of patients. This phase is characterised by lymphocyte apoptosis, impaired antigen presentation, reduced cytokine responsiveness, and increased susceptibility to secondary infections (4, 12). Importantly, these phases are not strictly sequential but often overlap, with elements of hyperinflammation and immunosuppression coexisting within the same patient at different anatomical and temporal levels.

The cytokine storm therefore reflects not only excessive immune activation but also a breakdown in regulatory control. Under physiological conditions, cytokines such as IL-6, IL-1β, TNF, and IFN-γ coordinate pathogen clearance, tissue repair, and immune homeostasis (13). In sepsis, however, this tightly regulated network becomes dysregulated, resulting in uncontrolled amplification of inflammatory signalling and systemic tissue injury. The consequent increase in vascular permeability, cellular dysfunction, and metabolic collapse underlies progressive multi-organ failure and, in severe cases, death (14, 15).

Within this immunological landscape, members of the IL-1 cytokine family exhibit functionally divergent roles. IL-1β and IL-18 primarily amplify inflammatory responses, whereas IL-37 and IL-38 function as endogenous immunoregulatory mediators that restrain excessive activation and facilitate resolution. IL-38 has been shown to suppress TNF, IL-23, IL-1β, and IL-6 production in human PBMCs derived from patients with SLE (16), and to reduce IL-22 and IL-17 levels in experimental autoimmune models (17). Among these, IL-37 and IL-38 are of particular interest because of their emerging roles as intrinsic regulators that fine-tune both innate and adaptive immune responses.

Importantly, the functions of IL-37 and IL-38 extend beyond simple suppression of inflammation. Rather than acting as constitutive inhibitors, accumulating evidence suggests that these cytokines operate as immune-metabolic rheostats that dynamically adjust the magnitude and quality of host responses according to the inflammatory and metabolic context (18). Similar to a rheostat that fine-tunes electrical current rather than merely switching it on or off, IL-37 and IL-38 modulate multiple interconnected pathways governing cytokine production, immune-cell activation, metabolic reprogramming, and tissue repair (19). In sepsis, where excessive inflammation and immune suppression frequently coexist and fluctuate over time, such rheostatic regulation may be particularly important for maintaining the balance between pathogen clearance and prevention of collateral tissue damage (20). Understanding how IL-37 and IL-38 contribute to this dynamic equilibrium may therefore provide new insights into the heterogeneous immunopathology of sepsis and identify opportunities for more precisely targeted immunomodulatory interventions.

The immunopathological roles of IL-37 and IL-38 in non-small cell lung carcinoma (21) and rheumatoid arthritis (22) have been well reviewed. However, despite increasing attention, their integrated roles in sepsis remain incompletely defined, particularly with respect to temporal dynamics, cellular specificity, and interaction with metabolic reprogramming. Current evidence remains fragmented, with most findings derived from preclinical systems or observational clinical studies.

Accordingly, the current mini-review synthesises current knowledge on IL-37 and IL-38 within the evolving framework of sepsis immunopathology, and highlights their potential relevance for precision immunomodulatory strategies, while recognising the limitations of existing translational evidence.

### Immunopathogenesis of sepsis: a dynamic continuum

Sepsis is initiated by recognition of pathogen- and damage-associated molecular patterns *via* pattern recognition receptors, including Toll-like and NOD-like receptors. This activates NF-κB and MAPK signalling pathways, resulting in rapid production of pro-inflammatory cytokines and chemokines (23, 24). While essential for pathogen clearance, excessive activation drives endothelial injury, vascular leakage, coagulation imbalance, and organ dysfunction, leading to adverse outcomes (25).

At the same time, compensatory anti-inflammatory mechanisms emerge, including regulatory cytokine production, expansion of Treg cells, and functional reprogramming of innate immune cells (26). As disease progresses, these mechanisms may predominate, leading to immune suppression characterised by reduced cytokine responsiveness, impaired phagocytosis, lymphocyte loss, and persistent epigenetic reprogramming of innate immunity (27).

Immunometabolism plays a central role in shaping this transition. Early immune activation is supported by glycolytic reprogramming driven by HIF-1α and mTOR signalling, whereas prolonged or late-stage disease is associated with oxidative phosphorylation, mitochondrial dysfunction, and reduced metabolic flexibility (28). Cytokines that couple immune signalling with metabolic regulation therefore occupy a central position in determining disease trajectory.

#### IL-37: coordinated suppression of inflammatory signalling and metabolism

### Molecular mechanisms

IL-37 exerts anti-inflammatory effects through both intracellular and extracellular mechanisms. Following caspase-1-mediated processing, IL-37 has been reported to interact with SMAD3 to suppress the transcription of pro-inflammatory genes, e.g. IL-27, IFN-γ, MIF, IL-8, RANTES. However, whether caspase-1-dependent cleavage is essential for IL-37 maturation and full biological activity remains incompletely understood, with evidence varying across different experimental models (29).

In parallel, extracellular IL-37 engages IL-18Rα together with the inhibitory co-receptor IL-1R8 (SIGIRR), leading to attenuation of downstream inflammatory signalling cascades (30). These intracellular and extracellular activities operate in a context- and environment-dependent manner, allowing IL-37 to regulate inflammation across multiple cellular compartments (31).

Beyond its direct effects on inflammatory signalling, IL-37 also influences cellular metabolism. It suppresses mTOR activity, limiting glycolytic reprogramming and favouring oxidative metabolic pathways. This metabolic shift is accompanied by reduced production of inflammatory mediators, including IL-18 and TNF (32). Through these combined actions, IL-37 links inflammatory signalling with metabolic state, supporting its role as a regulatory checkpoint within the immune response.

### Functional role in early sepsis

Circulating IL-37 levels are elevated in paediatric patients with sepsis compared with those with infection alone and non-infectious controls, as observed across multiple cohorts from different regions in China (33). Levels increase further in patients with septic shock, indicating an association with disease severity and mortality. Notably, IL-37 concentrations appear largely independent of infectious aetiology, showing comparable elevations across bacterial and non-bacterial infections as well as among infections caused by Gram-positive and Gram-negative pathogens. This pattern suggests that host immune dysregulation, rather than pathogen characteristics alone, is a key determinant of cytokine storm intensity.

In some clinical cohorts, admission IL-37 levels demonstrate prognostic value for 28-day mortality. Their predictive performance is second only to the SOFA score and exceeds that of procalcitonin and C-reactive protein, while remaining comparable to IL-6 (34). However, these findings are not consistent across all studies and should be interpreted in the context of cohort-specific variability.

Across adult, paediatric, and septic shock populations, circulating IL-37 correlates positively with inflammatory burden and organ dysfunction (33–35). This supports the view that IL-37 primarily reflects disease severity rather than exerting a directly protective effect in clinical settings. Despite its anti-inflammatory properties, elevated IL-37 levels are frequently associated with worse outcomes. This apparent contradiction is most plausibly explained by a compensatory yet insufficient response proportional to disease severity, rather than a direct pathogenic role. Accordingly, IL-37 is best interpreted as a context-dependent regulatory mediator rather than a primary driver of disease progression (12).

#### Mechanistic and experimental evidence

The role of IL-37 in sepsis has been examined extensively (33), using the caecal ligation and puncture (CLP) model (36). Administration of exogenous IL-37 reduces circulating chemokine levels in both *in vivo* and *in vitro* systems and inhibits pro-inflammatory M1 macrophage polarisation.

Recombinant IL-37 suppresses NF-κB- and MAPK-dependent cytokine production, including IL-6, CXCL1, and CCL2, while increasing IL-10 levels (33, 37). These effects are mediated, at least in part, through IL-1R8 (SIGIRR), which is required for effective IL-37 signalling. Functionally, IL-37 reduces leukocyte recruitment and limits pro-inflammatory M1 macrophage 1 polarisation without significantly altering total macrophage numbers. These immunomodulatory effects are associated with improved survival in experimental sepsis models (33, 38).

Taken together, these findings support a role for IL-37 as an endogenous regulator that integrates inflammatory signalling, immune cell function, and metabolic control. This activity is not uniformly beneficial. Sustained or excessive IL-37 expression may contribute to immune suppression, including impaired antigen presentation, reduced T cell activation, and induction of metabolic quiescence (12). Such effects may become detrimental in later stages of sepsis, when effective pathogen clearance is required.

#### IL-38: regulation of inflammasome activity and immune cell differentiation

IL-38 exhibits context-dependent biological activity and has been proposed to interact with IL-36R and potentially other receptors; however, its receptor landscape remains incompletely defined, which continues to limit mechanistic interpretation and translational development (39).

Circulating IL-38 is elevated in both adult and paediatric patients with sepsis compared with healthy controls, indicating that its expression is responsive to septic challenge. Notably, IL-38 levels are higher in survivors than in non-survivors, suggesting a potential association with favourable outcomes (40). In both adult and paediatric cohorts, IL-38 levels show inverse correlations with IL-6 and TNF, supporting a role in constraining excessive inflammatory responses (40). An additional inverse relationship between IL-38 and peripheral leukocyte counts has been reported in paediatric patients but not consistently in adults, raising the possibility that immune maturity influences IL-38-associated regulatory patterns during systemic inflammation (40).

The functional relevance of IL-38 has been investigated using the caecal ligation and puncture (CLP) model, which recapitulates key features of polymicrobial sepsis, including systemic inflammation across the blood, spleen, lungs, and peritoneal cavity (40). IL-38 expression is increased in the lung, spleen, peritoneal cavity, and serum of CLP mice, suggesting that IL-38 is upregulated as a compensatory response during sepsis and may confer protection. This is supported by the observation that neutralisation of IL-38 impairs host defence, as evidenced by reduced survival, worsened microbial clearance, and elevated circulating markers of organ injury, including alanine aminotransferase, aspartate aminotransferase, lactate dehydrogenase, and creatinine. In addition, IL-38 blockade is associated with increased expression of the pro-inflammatory cytokines IL-6, TNF, IL-17, IL-27, and chemokines CXCL1, and CCL2, together with upregulation of the chemokine receptors CXCR1 and CXCR3, while reducing survival and impairing bacterial clearance (40).

In addition, IL-38 blockade is associated with increased expression of the pro-inflammatory cytokines IL-6, TNF, IL-17, IL-27, and chemokines CXCL1, and CCL2, together with upregulation of the chemokine receptors CXCR1 and CXCR3, while reducing survival and impairing bacterial clearance (40).

This protective effect is associated with reduced levels of pro-inflammatory cytokines and chemokines, including IL-6, TNF, IL-17, IL-27, and CXCL1/2 across multiple organs (40), suggesting attenuation of cytokine storm. Interestingly, IL-10 levels are also reduced following IL-38 treatment, which may reflect a diminished requirement for compensatory anti-inflammatory responses in the context of attenuated inflammation. Conversely, blockade of IL-38 reverses these effects, leading to increased levels of the aforementioned cytokines and chemokines (40). In parallel, IL-38 enhances bacterial clearance, further supporting a functional role in host defence, although the underlying microbiological mechanisms remain to be clarified.

Mechanistically, these effects have been linked to increased Treg cell activity, promotion of Th2 polarisation, and suppression of effector T cell proliferation (41). The protective phenotype is lost following IL-38 neutralisation or Treg cell depletion, whereas exogenous IL-38 administration restores survival. Consistently, IL-38 increases IL-10 and TGF-β production in splenic cultures *in vitro* and exerts both preventive and therapeutic effects when administered either prior to or after CLP, while anti-IL-38 antibodies markedly increase mortality (41).

However, an apparent discrepancy exists regarding IL-10 regulation, as reduced levels have also been reported in other *in vivo* settings (40). This divergence may reflect context-dependent effects of IL-38, differences between *in vitro* and *in vivo* systems, or stage-specific immune dynamics during sepsis progression. Further studies are therefore required to clarify the temporal and compartment-specific regulation of IL-10 in response to IL-38 modulation.

IL-38 and its associated signalling components, including IL-36R (IL1RL2), are upregulated in CD4^+^CD25^+^ regulatory T (Treg) cells following lipopolysaccharide (LPS) stimulation *in vitro* (41). Functionally, IL-38 enhances the expansion and suppressive activity of Treg cells, and depletion of Treg cells significantly impairs host immunity and reduces survival, highlighting their essential role in IL-38-mediated protection (41). In addition, IL-38 promotes the production of anti-inflammatory cytokines such as IL-10 and TGF-β (41), while reducing pro-inflammatory cytokines (IL-6, TNF, IL-17, IL-27) and chemokines CXCL1 and CXCL2 across multiple organs (40).

Collectively, these findings suggest that IL-38 attenuates the cytokine storm partly through Treg-dependent mechanisms by enhancing anti-inflammatory responses and suppressing excessive pro-inflammatory signalling. This immunoregulatory effect may also contribute to a shift in immune cell polarisation, promoting M2 macrophage differentiation over the pro-inflammatory M1 phenotype.

At the molecular level, IL-38 inhibits activation of the NLRP3 inflammasome and reduces macrophage apoptosis, thereby limiting IL-1β production (42), potentially *via* suppression of NF-κB signalling (43). In addition, IL-38 modulates adaptive immunity by inhibiting Th17 differentiation and enhancing Treg cell activity, contributing to immune homeostasis (44).

Collectively, these findings indicate that Treg cells are essential for IL-38-mediated protection against sepsis, with IL-38 likely acting in both autocrine and paracrine manners to regulate Treg cell responses. Despite these advances, most clinical evidence for IL-38 remains observational and comparatively limited relative to IL-37, highlighting a clear gap in translational maturity between these cytokines.

### Integrated and stage-independent roles

IL-37 and IL-38 are best understood as components of a shared regulatory network within the IL-1 cytokine family rather than as mediators confined to discrete phases of sepsis. IL-37 is closely associated with suppression of NF-κB- and MAPK-driven inflammatory signalling and modulation of metabolic pathways through mTOR, particularly during periods of heightened inflammatory activation. IL-38, in contrast, is more prominently linked to regulation of inflammasome activity and modulation of adaptive immune responses.

**Figure 1:**
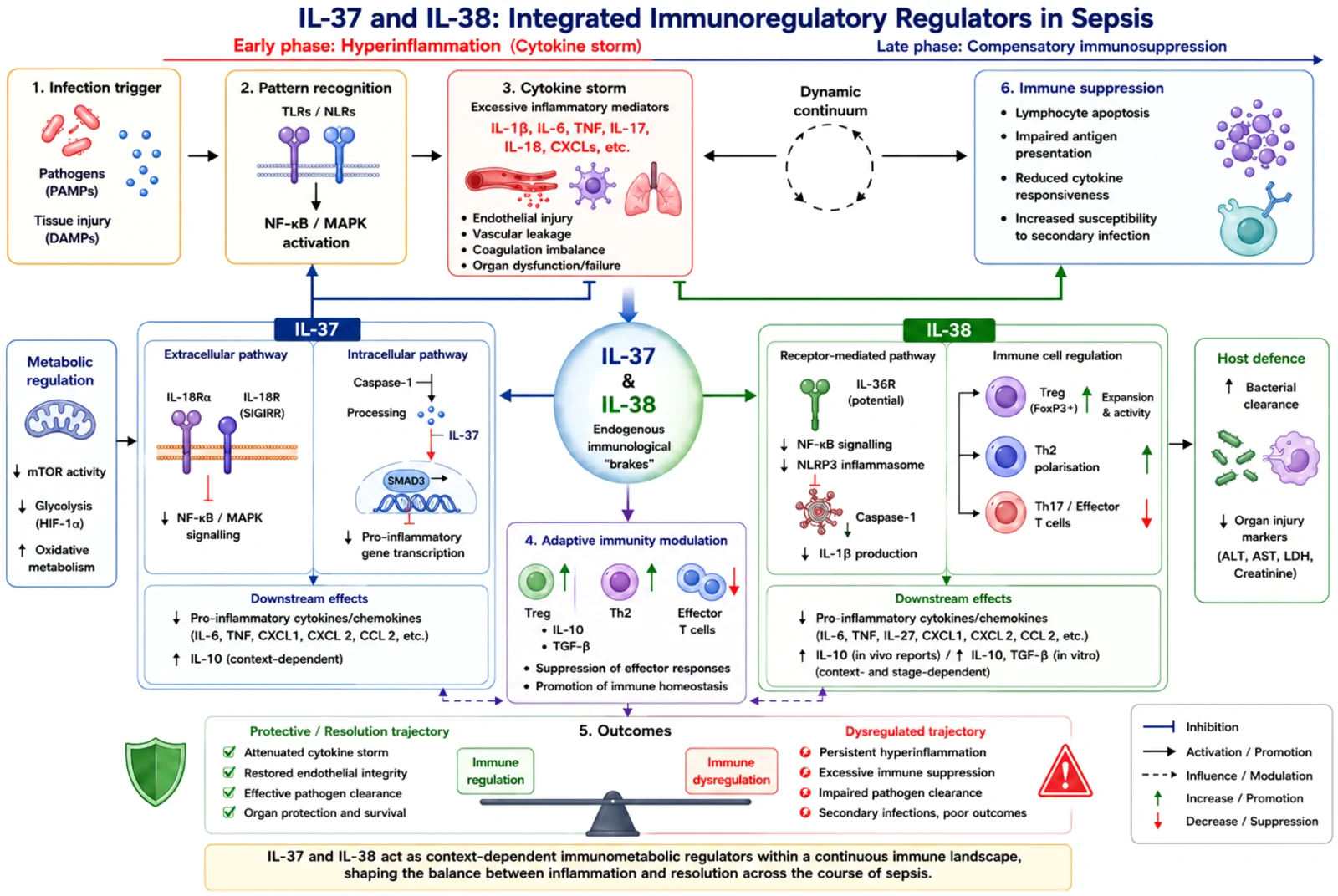
Integrated immunoregulatory roles of IL-37 and IL-38 in sepsis. Schematic of sepsis progression from pathogen recognition and NF-κB/MAPK-driven cytokine storm to immune suppression and organ dysfunction. Excessive production of IL-1β, IL-6, TNF, and chemokines leads to endothelial injury, vascular leakage, and multi-organ failure, followed by compensatory but dysregulated immunosuppression characterised by lymphocyte loss and impaired antigen presentation. IL-37 suppresses NF-κB/MAPK signalling via intracellular SMAD3 and IL-18Rα/IL-1R8 engagement, and modulates immunometabolism through mTOR inhibition. IL-38 inhibits inflammasome activation and IL-1β production while enhancing Treg activity, suppressing Th17 differentiation, and reducing effector T cell proliferation. Both cytokines attenuate systemic inflammation and support bacterial clearance. The image highlights IL-37 and IL-38 as context-dependent immunoregulatory regulation within a dynamic immune–metabolic continuum, where their balance influences progression towards resolution or persistent dysregulation.

Whether these cytokines act sequentially or as part of a continuously operating regulatory circuit remains unresolved. Current evidence favours a model in which their relative influence shifts according to the prevailing immune-metabolic state rather than fixed temporal stages. Their functions overlap, and imbalance in either direction may contribute to divergent pathological outcomes, including persistent inflammation or immune suppression. A schematic representation of these interactions is provided in **Figure 1** to illustrate their coordinated roles within the broader immune landscape of sepsis.

### Clinical and translational implications

IL-37 and IL-38 have emerged as promising biomarkers and potential therapeutic targets in sepsis; however, meaningful clinical translation remains challenging. The repeated failure of cytokine-targeted interventions underscores the complexity of sepsis biology and highlights the importance of timing, patient stratification, and underlying immune heterogeneity.

Future strategies will likely need to move beyond static biomarker assessment towards approaches informed by longitudinal immune profiling. In this setting, the dynamic nature of immune responses may be more accurately captured, allowing interventions to be tailored to evolving disease states. Key challenges include defining appropriate therapeutic windows, avoiding exacerbation of immune suppression, and identifying biomarkers that reliably reflect shifting immune–metabolic conditions.

### Limitations and future directions

Several limitations continue to constrain progress in this field. Longitudinal human data remain limited, particularly for IL-38, and receptor interactions and downstream signalling pathways are not yet fully characterised. In addition, much of the current understanding is derived from preclinical models that do not fully recapitulate the clinical heterogeneity of sepsis. While their biological potential is stage-independent, our current clinical evidence is heavily biased toward the acute presentation phase.

Further work integrating single-cell approaches, immunometabolomics, and time-resolved immune profiling will be important for clarifying how IL-37 and IL-38 function across different stages and patient subsets. Such studies may help define their roles within the broader immune network and support the development of more targeted therapeutic strategies.

## Conclusions

IL-37 and IL-38 are emerging regulators within the IL-1 cytokine family that integrate inflammatory and metabolic signalling in sepsis. Rather than operating as stage-specific mediators, they appear to act in a context-dependent manner, shaping the balance between inflammatory activation and immune resolution. A clearer understanding of their temporal and functional dynamics will be essential if their therapeutic potential is to be realised, particularly within precision-based approaches to sepsis management.
